# Molecular Methods for the Detection of *Toxoplasma gondii* Oocysts in Fresh Produce: An Extensive Review

**DOI:** 10.3390/microorganisms9010167

**Published:** 2021-01-13

**Authors:** Iva Slana, Nadja Bier, Barbora Bartosova, Gianluca Marucci, Alessia Possenti, Anne Mayer-Scholl, Pikka Jokelainen, Marco Lalle

**Affiliations:** 1 Department of Microbiology and Antimicrobial Resistance, Veterinary Research Institute, Hudcova 296/70, 621 00 Brno, Czech Republic; slana@vri.cz (I.S.); bartosova@vri.cz (B.B.); 2 Department of Biological Safety, German Federal Institute for Risk Assessment (BfR), Max-Dohrn-Str. 8-10, 10589 Berlin, Germany; nadja.bier@bfr.bund.de (N.B.); Anne.Mayer-Scholl@bfr.bund.de (A.M.-S.); 3 Unit of Foodborne and Neglected Parasitic Diseases, European Union Reference Laboratory for Parasites, Department of Infectious Diseases, Istituto Superiore di Sanità, viale Regina Elena 299, 00161 Rome, Italy; gianluca.marucci@iss.it (G.M.); alessia.possenti@iss.it (A.P.); 4 Laboratory of Parasitology, Infectious Disease Preparedness, Department of Bacteria, Parasites & Fungi, Statens Serum Institut, Artillerivej 5, DK-2300 Copenhagen S, Denmark; PIJO@ssi.dk

**Keywords:** *Toxoplasma gondii*, oocyst, ready-to-eat (RTE) salad, fresh produce, detection, toxoplasmosis, zoonosis, foodborne parasites

## Abstract

Human infection with the important zoonotic foodborne pathogen *Toxoplasma gondii* has been associated with unwashed raw fresh produce consumption. The lack of a standardised detection method limits the estimation of fresh produce as an infection source. To support method development and standardisation, an extensive literature review and a multi-attribute assessment were performed to analyse the key aspects of published methods for the detection of *T. gondii* oocyst contamination in fresh produce. Seventy-seven published studies were included, with 14 focusing on fresh produce. Information gathered from expert laboratories via an online questionnaire were also included. Our findings show that procedures for oocyst recovery from fresh produce mostly involved sample washing and pelleting of the washing eluate by centrifugation, although washing procedures and buffers varied. DNA extraction procedures including mechanical or thermal shocks were identified as necessary steps to break the robust oocyst wall. The most suitable DNA detection protocols rely on qPCR, mostly targeting the *B1* gene or the *529* bp repetitive element. When reported, validation data for the different detection methods were not comparable and none of the methods were supported by an interlaboratory comparative study. The results of this review will pave the way for an ongoing development of a widely applicable standard operating procedure.

## 1. Introduction

Toxoplasmosis is a zoonotic parasitic disease caused by the protozoan *Toxoplasma gondii* (*T. gondii*; [1]). The clinical manifestations of toxoplasmosis in humans, including congenital, cerebral and ocular toxoplasmosis, cause a substantial disease burden worldwide [2,3] Moreover, *T. gondii* can also cause clinical disease in its animal hosts, resulting in major losses in livestock industry and lower welfare for the affected animals [1].

It has been estimated that 42–61% of acquired toxoplasmosis cases are foodborne [4]. The food- and waterborne transmission routes of *T. gondii* are numerous, including ingestion of infective tissue-dwelling stages of the parasite in raw or undercooked meat of infected animals and ingestion of oocysts, shed by infected felines and sporulated in the environment, in contaminated water or food, such as fresh produce (fruits, vegetables, and juice) [5]. 

Although *T. gondii* is a highly prioritized zoonotic foodborne pathogen in Europe and worldwide [6,7], it is not systematically controlled. Evaluation of *T. gondii* oocyst contamination of fresh produce, such as ready-to-eat (RTE) salad leaves, is an unfilled need of both the public health sector and food industry, especially with an increasing consumer preference for these food items [8]. Although scientific literature has reported an association between the consumption of unwashed fresh produce and *T. gondii* infection, the relative importance of this infection source remains unknown [5] and outbreak investigations are scarce. The typically low numbers of *T. gondii* parasites in food matrices makes detection challenging, and at present, no specific regulations or ISO standards are available for detection of *T. gondii* in any food matrix [5]. Thus far, ISO standards have been developed to detect few other foodborne protozoan parasites in fresh produce. The ISO standard method (ISO 18744:2016) for the detection of *Cryptosporidium* spp. and *Giardia* spp. on leafy greens and berry fruits is based on visual detection by immunofluorescence microscopy and is not amenable to high-throughput testing. In order to address food safety risk assessment challenges typical for foodborne protozoan parasites, it is essential that the testing moves to standardised molecular assays, similar to e.g., US FDA—BAM 19b for “Molecular Detection of *Cyclospora cayetanensis* in Fresh Produce Using Real-Time PCR” [9].

Molecular methods for detecting *T. gondii* oocyst contamination have been described in the literature, but a widely applicable method remains to be defined. To provide a solid basis for method development and standardisation, we performed an extensive literature review and multi-attribute assessment of the described and currently used methods.

## 2. Materials and Methods

**Figure 1:**
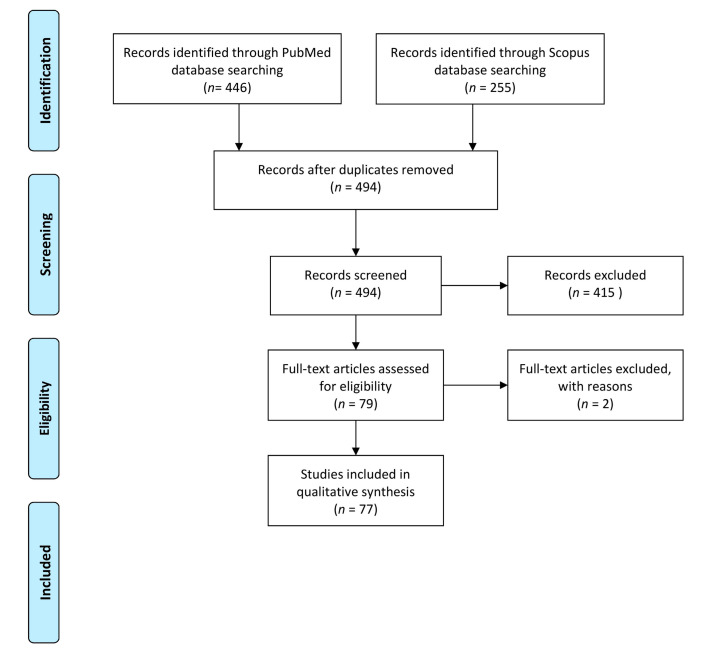
PRISMA flow diagram of the search strategy steps for the literature review.

We searched two online databases, PubMed and Scopus, for all potentially relevant records on molecular methods applied to detect *T. gondii* oocysts, irrespective of the matrix, following PRISMA (Preferred Reporting Items for Systematic Reviews and Meta-Analyses) guidelines where applicable [10] (Figure 1). The search terms were grouped into 14 combinations (Supplementary File S1). The databases were searched for records in English that were published up to 12 February 2020. Publications were initially screened by three independent reviewers for eligibility based on title and abstract. Then, records were excluded if they were: (i) letters, editorials, notes, comments, and reviews; (ii) studies describing methods not applicable to *T. gondii* or (iii) methods using reagents that are not widely available (e.g., antibodies). Full texts of the records were screened by six independent reviewers. Records were excluded at this stage if (i) full text was unavailable or if (ii) the study did not describe methods applicable to molecular detection of *T. gondii* oocysts. 

For data extraction, we focused on three key steps of the detection: oocyst recovery, DNA extraction and DNA detection. From each eligible record, data were extracted in predefined tables (Supplementary File S1). For the oocyst recovery step, only data extracted from studies using fresh produce as matrix were included in the final analysis and assessment of the data. Experimental contamination (spiking) studies were considered to provide information of great relevance for the oocyst recovery step, as they are performed under controlled experimental conditions. Data on the three main steps were analysed independently. 

The extracted data were complemented with the output from a survey conducted in February 2020 among 24 expert laboratories with experience in *T. gondii* detection in food and non-food matrices [11]. The questionnaire collected information about current practices, details and facilities for molecular testing for *T. gondii* in different matrices, as well as expert opinions on methods for molecular detection of *T. gondii* oocysts.

## 3. Results

Of the identified 494 records, 77 studies were included in the review (Figure 1 and Supplementary File S1). The matrices tested were: water (27 studies), edible and non-edible bivalves (16 studies), soil (15 studies), fresh produce (14 studies), faeces (12 studies), and other matrices (3 studies).

Thirteen records reported on analytical procedures for molecular detection of *T. gondii* in fresh produce. Of these, nine described method evaluation using fresh produce that was spiked with oocysts: eight with sporulated *T. gondii* oocysts and one with *Eimeria papillata* oocysts [12,13,14,15,16,17,18,19,20] (Table 1). The other four papers described surveys (Table 2) [21,22,23,24]. The fresh produce types tested, the amount of tested matrix, sample preparation and spiking protocols, as well as the number of oocysts used for spiking varied considerably between the studies (Table 1 and Table 2).

In the included spiking studies, the matrices used were berries (strawberries, raspberries, blackberries, blueberries and cranberries), leafy greens (basil, lettuce, spinach, cilantro, dill, mint and parsley) and other vegetables (radish, thyme and green onions) (Table 1). The sample amount used ranged from 10 g to 60 g (Table 1). For spiking, six studies used a dripping method (pipetting the oocyst suspension onto the matrix surfaces, mimicking vegetable contamination by irrigation), while two studies used an immersion method (i.e., the material was immersed in water containing a known amount of oocysts) (Table 1). A post-spiking incubation to allow oocyst adherence after applying a dripping method ranged from 30 min to overnight, and temperatures used were room temperature and +4 °C. Oocysts of *T. gondii* are highly resistant to environmental conditions but do not multiply in the environment; consequently, the first step for parasite detection requires parasite enrichment by parasite recovery and concentration. All procedures for oocyst recovery from leafy greens involved washing and pelleting of the washing eluate by centrifugation. Additional steps reported prior to centrifugation included overnight flocculation using CaCO_3_ solution, filtration through cellulose ester membrane and flotation using Sheather’s sucrose solution (Table 1). Limited information on the impact of these additional steps on recovery rate could be extracted. Despite the fact that it was not considered in our literature review effort, it is worth mentioning that even the use of a immunomagnetic separation (IMS) step with non-commercially available in-house anti-*T. gondii* oocyst monoclonal antibodies did not result in any improvement of the recovery rate (as quantified by qPCR) [13]. Filtration, flocculation and flotation might partially replace centrifugation, which can be a limiting factor when using large volumes of wash buffer or a large number of samples [16,17,18,19]. Although flocculation and flotation might also reduce soil particles and other contaminants that could potentially inhibit DNA amplification [16,17,18,19], one study underlined that if flotation is used, residual Sheather’s solution could inhibit downstream PCR reactions [17]. Washing of vegetables was performed either manually, using an automatic horizontal orbital shaker from 15–30 sec to 60 min, or by stomaching (Table 1). The most commonly used washing buffers were aqueous solutions of 1 M glycine pH 5.5 (four studies) or 0.1–1% Tween 80 (four studies) in the range of 4–6 mL of washing buffer per gram of sample (Table 1). Two studies compared different washing methods: stomaching of leafy herbs contaminated with *E. papillata* oocysts with 1 M glycine pH 5.5 buffer provided a higher recovery percentage than horizontal orbital shaking did, whereas for spinach spiked with heat-inactivated *T. gondii* oocysts, manual shaking with 0.1% Tween 80 was more effective than stomaching [15,17]. One of the studies highlighted that washing buffers containing a surfactant or detergent are not recommended for stomaching, since the bubbles produced during the homogenisation seemed to interfere with oocyst recovery [17]. An evaluation of washing buffers was reported in two studies, both using leafy greens but different washing protocols (manual washing vs automatic shaking or stomaching): Both studies reported that 1 M glycine pH 5.5 performed better than PBS [12,17]. Among the questionnaire survey participants who reported testing fresh produce, stomaching, manual washing and pelleting by centrifugation were the most common sample processing techniques [11]. The reported washing buffers were similar to those reported in the published literature. In the six prospective surveys included, a larger variety of leafy greens were tested, including mixed salads (Table 2). One study [19] used a large amount of the tested sample, up to 1 kg, as well as a large volume of washing buffer (≥2 L) followed by flocculation. In four studies, the amount of tested samples ranged from 35 g to 100 g, with an average volume of washing buffer of 2 mL/g of sample [18,21,22,24]. The washing buffers used in these four studies contained either Tween-80 (0.1–1%) or glycine. Three studies reported washing using an automatic shaker for 15–20 min or up to 2 h, followed by centrifugation [17,19,22]. Additionally, in the largest survey with over 1000 samples, 35 g of sample was tested, washing was done with 200 mL of 1 M glycine pH 5.5 using an orbital shaker or stomacher, and oocysts were recovered by centrifugation and flotation with Sheather’s sucrose solution [21]. Prior to DNA extraction, a step to break the wall of the oocysts was included in all spiking studies (Table 1). Bead-beating (BB) using a commercial mix of beads in combination with a high-speed mechanical homogenizer, with single or double cycles at speeds in the 4–6.5 m/s range for 30 s to 2 min, were used in four studies [12,14,19,20]. The freeze and thaw (FT) method, with 1 to 10 cycles, temperature ranges from −196 °C to 100 °C, and incubation times of 1–5 min, was used in four studies [13,15,16,18]. FT was as effective as to ultrasound (US), when compared to no pre-treatment [16]. In two studies, US or incubation with proteinase K at 56 °C was used as an additional step after FT cycles [13,17]. Three spiking studies on non-vegetable matrices evaluated the performance of different DNA extraction procedures for *T. gondii* oocysts including BB and/or FT [25,26,27] (Table 3). 

Use of commercial kits, including sample homogenisation by BB, performed better than the procedure using sedimentation/flotation in combination with FT followed by in-house phenol-chloroform extraction and the DNA extraction kit without BB, even when an additional step using glass beads was included [25]. In one study, the combination of BB, FT and proteinase K treatment together with a commercial DNA extraction kit showed higher sensitivity than vortexing and BB followed by DNA extraction using another commercial kit [26]. One study suggested that increasing the number of FT cycles did not enhance oocyst DNA detection and may have resulted in decreased sensitivity due to DNA degradation [27]. According to the questionnaire results, BB was used in the majority of the participating laboratories for testing of fresh produce [11]. When all the included studies where considered, 15 reported on the use of BB for DNA extraction from *T. gondii* oocysts [14,19,20,24,25,26,28,29,30,31,32,33,34,35] (Supplementary File S1) with two commercial kits most frequently used (Supplementary File S1). FT associated with silica spin-column kits was reported in 58 studies (Supplementary File S1). Concerning molecular detection, conventional PCR (cPCR) was used in 17 of the 77 reviewed studies (22%), nested or semi-nested PCR in 20 studies (26%) and two papers reported using loop-mediated isothermal amplification (LAMP) (Supplementary File S1). For the cPCR assays, most studies targeted the *B1* gene or the *529* bp repetitive element (*529RE*) (Table 4). Six studies compared the sensitivity of cPCR targeting *B1* vs. *529RE*, expressed as the limit of oocysts providing a positive amplification (Supplementary File S1). For fresh produce, *B1*-cPCR was shown to be 10 times more sensitive than *529RE*-cPCR [16], with a limit of detection (LoD) of 10 and 100 oocysts/heads of lettuce, respectively. For soil and faeces, the results were the opposite [36,37,38]. The sensitivity of the *529RE*-cPCR is also affected by the efficiency of the DNA extraction method [25] and reducing amplicon size was beneficial [38]. Sensitivity appeared generally higher in water or DNA-poor matrices than in complex matrices [e.g., 26]. 

Assays relying on qPCR accounted for almost 50% of studies (38 studies) and were mainly qualitative, with Taqman assays targeting the *529RE*, which was the most often applied (Table 5 and Supplementary File S1).

**Figure 2:**
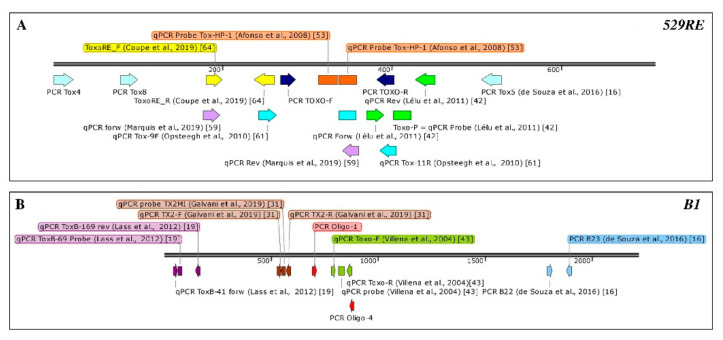
Graphical representation (using Snap Gene Viewer 5.0.7 software, GSL Biotech LLC, US) and localisation of primers and probes reported in Table 4 and Table 6 and used in cPCR and qPCR assays to target: (**A**) the *529 RE* (Accession number FJ656209.1) and (**B**) the *B1* genes (Accession number AF179871.1).

Two qPCR assays targeting the *529RE* [26,42] and two assays targeting the *B1* gene [19,43] were most commonly used (Table 6 and Figure 2). One study reported that the addition of MgCl_2_ (up to 5 mM) improved the performance of a *B1*-qPCR assay [31]. Comparing the sensitivity of the different assays was challenging due to differences in spiking protocols and reporting of the LoD.

In 13 papers reporting analytical procedures for molecular detection of *T. gondii* in fresh produce (Table 1 and Table 2), three studies used cPCR [12,16,18], whereas DNA detection was done by qPCR in 10 studies, either using Taqman-assays (seven studies) [13,14,15,19,20,23,24] or High Resolution Melting (HRM) analysis (three studies) [17,21,22]. The qPCR assays targeted either the multicopy genes *18S-rDNA* (two studies) [17,21], *B1* (three studies) [19,22,23] or the *529RE* (four studies) [13,14,15,20], and one assay was a multiplex qPCR targeting both *B1* gene and the *529RE* [24]. None of the studies reported a full validation process or included a ring-trial to assess the reproducibility of the assay.

One *B1*-qPCR assay used for the analysis of fresh produce provided a LoD of 100 oocysts/per radish [19]. Sensitivity of less than 1 oocyst/g of fresh produce was reported for two different *529RE*-qPCR assays [13,14,20] (Table 1 and Table 6). The analytical and diagnostic performance of the endpoint *529RE* cPCR using the primer Tox5 and Tox8 [38] and two *529RE*-qPCR assays, one of which used the Tox-9F and Tox-11R primers and the probe Tox-TP1 (originally described in Reischl et al., 2003, Opsteegh et al., 2010) [56,61], were evaluated in a recent publication for *T. gondii* DNA detection in pork [60]. Both the *529RE*-qPCRs were shown to provide similar sensitivity and specificity, but with a higher sensitivity than the corresponding cPCR [60]. For some studies, performance characteristics of qPCR assays were reported [20,23,29,60,62,63,64]. In particular, in Temesgen et al. 2019 [20], the *529RE* Taqman assay [61] applied to fresh produce (berries) was evaluated for specificity, efficiency, linearity, LoD, repeatability, intermediate precision, and robustness. The original assay was further improved with the use of MGBEQ-labelled probe instead of BHQ1. Nine studies [23,24,29,43,44,45,49,54,58] reported the use of internal amplification controls (IAC) in the qPCR assay, including among others a competitive IAC (CIAC) [45] and synthetic targets [23,24].

## 4. Discussion

Detection of *T. gondii* in vegetables is challenging due to the low sensitivity of existing detection methods. This also holds true for other foodborne parasites (e.g., *Cryptosporidium* spp. and *Giardia duodenalis*) [65]. As oocysts of *T. gondii* are highly resistant to environmental conditions and do not multiply in the environment, oocyst recovery from fresh produce is the first and key step to enable successful detection. Molecular detection must then rely on efficient DNA extraction from the robust oocysts, together with a reduction of possible contaminants that could inhibit the DNA amplification. Finally, amplification must be specific and sensitive to detect DNA from low numbers of oocysts, ideally a single oocyst, avoiding any cross-amplification with closely related species. 

As shown in this review, many different methods have been described for each step of the molecular detection of *T. gondii* oocysts and different combinations of them have been used to analyse fresh produce as well as other matrices. This variability, which was also evident in the results of the questionnaire survey [11], prevents a direct comparison of the studies to identify the most promising method for a sensitive and reliable detection of *T. gondii* oocysts in fresh produce (as well as in other matrices). Although specific characteristics of different vegetable matrices can interfere with oocyst recovery due to e.g., trapping and adhesion force and, later on, with molecular detection (i.e., different concentrations of PCR inhibitors), the overall molecular detection procedure should be harmonised and standardised. The oocyst recovery step from fresh produce is particularly important but challenging to standardise due to a large variability in the reported methods (e.g., washing procedure, washing buffers and oocyst concentration). For instance, stomaching with an appropriate setting of homogenisation power and speed to account for brittleness of the vegetable samples, would be a fast procedure to apply for large scale analysis and easy to standardize. Due to the presence of high amounts of natural detergents in some types of fresh produce (e.g., saponins in spinach), the use of washing buffers with detergent (i.e., Tween-80) might not be recommended as they could exacerbate foaming and potentially trap oocysts in the foam, thus lowering the recovery rate. The 1 M glycine solution is potentially the buffer of choice, as it is inexpensive and did not generate an excess of debris during stomaching of lettuce as the sample matrix [66], which could eventually interfere with downstream oocyst concentration and DNA extraction. Although oocysts concentration by centrifugation might be time consuming and require a centrifuge, other procedures might be more complicated or less efficient. For instance, flocculation of water samples with Fe_2_(SO_4_)_3_ resulted in PCR inhibition [65]. The risk of oocyst loss following NaNO_3_ flotation was highlighted in one study on soil samples [34], suggesting that NaNO_3_ flotation is suitable when oocyst contamination is ≥103/40 g soil. One paper discussed that while flocculation is simple and inexpensive, filtration is more robust for processing turbid wastewater (and possibly the washing suspensions of vegetables), and PCR inhibitors appeared to be eliminated by using 1-μm pore-sized polyethersulfonate membrane filters [43]. Additionally, filtration would be preferable when large volumes (litre) of a sample need to be processed.

The reported DNA extraction protocols substantially differ in their approach to break the robust oocyst wall (FT, US and BB), whereas further DNA purification and clean up from inhibitors are mostly performed using silica-column-based DNA extraction kits. Although FT does not require expensive equipment, in contrast to the use of a bead beater, the choice of the most promising and efficient FT procedure is difficult due to the large variability of settings applied in different studies (e.g., length and number of reported freeze and thaw cycles were quite different). Moreover, the requirement of several cycles of FT is time consuming especially when a large panel of samples is tested. According to most of the manufacturer’s protocols, kits using pre-packed silica spin columns allow the use of only a fraction of the supernatant obtained from the initial sample lysis per single extraction. This might lead to a considerable loss of material and reduction of the final assay sensitivity, as either only a portion of the original sample is used for the DNA extraction step, or might require multiple DNA extraction from the same sample with consequent increase in assay time and costs. Commercial kits including a mechanical disruption step (e.g., BB) have already been successful in detecting *Hammondia* spp. and *T. gondii* oocysts by PCR with a high sensitivity [25]. Furthermore, they have the advantage of using larger sample volumes without substantial adaptation of the kit that are loaded with a silica matrix onto empty columns and could, therefore, favour a higher assay sensitivity. Whether the performance of different commercial kits based on BB is comparable or not was not specifically assessed in any of the papers included in this study, but might be presumed by the comparability of two kits tested in Herrmann et al., 2011 (specifically NucleoSpin Soil from Marcherey-Nagel vs ZymoResearch fecal DNA Kit from Zymo) [25]. However, since available kit formulations and producers might change over time and in different countries, kit performance should always be evaluated prior to a study, in order to select the most suitable kit.

For the purpose of this review, we did not further consider nested-PCR and LAMP (loop-mediated isothermal AMPlification) assays as suitable for routine testing of fresh produce. Despite their higher sensitivity and specificity compared to conventional PCR, both techniques suffer from a high risk of background and cross-contamination, and nested PCR requires two consecutive rounds of amplification. Concerning the reported molecular assays, qPCR targeting either the *B1* gene and/or the *529RE* both provide a very high sensitivity, due to multiple copies of both targets in the *T. gondii* genome. Although double-strand DNA-intercalating fluorescence dyes (e.g., SYBR Green) combined with melting curve analysis (MCA) are relatively cost beneficial and easy to use, dual-labeled TaqMan probes have the advantage of combining detection with confirmation of the amplification products without the need for further amplicon sequencing. It should be noted that the specificity of the amplification product can be of concern, especially when targeting *529RE*, due to potential cross amplification with parasites closely related to *T. gondii* (i.e., *Hammondia hammondi*, *Sarcocystis* spp. *Neospora caninum*) [38,60].

PCR inhibitors are important confounders that must be addressed in any PCR-based detection effort. Molecular detection of pathogens in food can be challenging due to a large variety of PCR inhibitors that can be co-extracted with DNA. Especially, DNA extracts from pelleted washing suspensions of plant-based food may contain diverse PCR-inhibiting substances from debris of the plants themselves (e.g., phenols, polyphenols, polysaccharides), but also from residual soil or irrigation water components (e.g., humic and fulminic acids) [67]. Depending on the food matrix and type and mechanism of inhibitory substances, different strategies can be evaluated to decrease their concentration in the sample or to reduce their inhibitory effect by e.g., using less-sensitive polymerases or specific PCR additives (e.g., BSA, DMSO) [67]. It should be noted that, for the detection of PCR inhibitors and to exclude false-negative results, the use of an internal amplification control (IAC) is mandatory for diagnostic PCR detection of foodborne pathogens according to CEN/ISO 22174 [68]. Competitive IACs are synthetic oligonucleotides that are amplified with the same set of primers as the target gene. Although they are amplified under the same conditions and thus mimic the amplification of the target gene, they also have a stronger potential to reduce the assay sensitivity and may require more optimization work [69]. As low sensitivity and inhibition is already an issue when analysing fresh produce for *T. gondii*, we rather propose the application of non-competitive IACs, ideally as a synthetic sequence with no homology with either the target parasitic DNA or with the matrix, as for example used in the US FDA—BAM 19b for “Molecular Detection of *Cyclospora cayetanensis* in Fresh Produce Using Real-Time PCR” [9]. As these non-competitive IACs are amplified with a different set of primers, they can universally be applied in different PCR detection systems and have the advantage of generally not competing with the target amplification, when used in low concentrations and with limiting primer concentrations. 

We would like to stress that for any published qPCR assay, it is important to report the performance characteristics according to the MIQE guidelines [70,71,72]. This includes: (i) use of an IAC to check for PCR inhibition; (ii) preparation of a standard curve (10-fold serial dilution of at least five template concentrations) with background matrix (e.g., pelleted washing suspensions from uncontaminated food matrix); (iii) evaluation of amplification efficiency and linearity with a R^2^ value (ideally ≥0.98); (iv) determination of the LoD95%, supported by spiking studies. 

A standardized procedure to be applied for the detection in fresh produce would not only be desirable for *T. gondii* but also for other foodborne protozoan parasites. Of course, implementation of slightly different methods might be necessary to reliably detect the target parasite. The problems associated with the availability of a large number of laboratory methods for pathogen detection are manifold. If prevalence data are not comparable from different regions or countries, they might result in inaccurate risk assessment conclusions. For instance, if quantification is required, it is necessary to define the quantified target (DNA amount, number of oocysts, target copy number) as well as a standardized and harmonized procedure to convert this to equivalent numbers of oocysts (indeed oocysts load is the data that food stakeholders might expect). This is exacerbated by the fact that the large majority of published methods are not or insufficiently validated. Although ISO standards for the validation of parasitic methods are currently not available, method validations can be based on a number of available documents [70,73,74,75,76,77,78,79].

Noteworthy, none of the articles reviewed reported on any attempt to evaluate the applied methodology through an inter-laboratory comparison. Results of ring trials are an important indicator for the inter-assay precision (reproducibility) of a method and an essential step for the better understanding of the method characteristics. As already described for the validation of microbiological methods in the food chain, inter-laboratory comparisons should also be performed as part of the validation of parasitological methods. In light of the increasing internationalisation of food supply chains, the need for conclusive data to better understand food-borne transmission or to provide a solid basis for risk assessments, has increased. For this, robust, validated and standardized laboratory methods for the detection of contamination of food sources with a high level of confidence are essential. This is especially important for *T. gondii*, a highly prioritized zoonotic foodborne pathogen, where laboratories are currently using a multitude of different diagnostic approaches.

## Figures and Tables

**Table 1:** Published methods for detection of *Toxoplasma gondii* in fresh produce that have been evaluated using spiking experiments.

Type of Spiking	Matrix (Grams)	Spiking Level (oo)Cysts	Time after Spiking	Processing Method	Washing Buffer (mL)	Recovery (%) (Quantitative Evaluation) ^###^	Pre-Treatment before DNA Extract	DNA Extraction	Detection Method (Target Gene)	Amplicon Size (bp)	LoD (Oocysts)	Reference
dripping	basil (25)	10^2^	ON at 4 °C	wash by hand shaking for 15 s, hand rubbing for 30 s, shaking vigorously for 15 s and centrifugtion	six different buffers (200 mL) ^#^	NR	BB (5.5 m/s for 30 s)	Fast DNA Spin for Soil kit	PCR (*529 RE*)	529	depend on the washing buffer ^##^	[12]
	basil (30) raspberries (30)	5 to 10^4^	2 h at RT	wash by automatic shaker (80 rpm 10 min), centrifugation, IMS Toxo	1 M glycine pH 5.5 (200 mL)	basil: 0.2% microscopy; 35% qPCR raspberry: 2% microscopy; 29% qPCR	FT 6× (−80 °C for 5 min/95 °C for 5 min) and US (1 min at 37 Hz)	InstaGene Matrix	qPCR Taqman (*529 RE*)	81	Basil: <33/g Raspberries: <33/g	[13]
				wash by automatic shaker (80 rpm 10 min), centrifugation		basil: 35% qPCR raspberries: 2.5% qPCR					Basil: <1/g Raspberries: <1/g	
	baby lettuce (50)	25, 50, 100 for 50 g	ON at 4 °C	wash by stomaching ^b^ and centrifugation	1 M glycine pH 5.5 (200 mL)	NR	BB 2× (6.0 m/s for 40 s)	FastPrep for soil kit	LAMP (*529 RE*)	NA	0.5/g or 5/mL	[14]
		5, 10, 50 in 800 µL (pellet)	ON at −20 °C						qPCR_Taqman (*529 RE*)	163		
	spinach (10)	10^1^ to 10^4^	2 h at RT	wash by stomaching and centrifugation	0.1% Tween 80 (100 mL)	≤30% by microscopy (IMS/membrane filtration)	FT 1× (4 min in N_2_ and 4 min 100°C)	DNeasy Blood and Tissue Kit	nPCR (*rDNA 18S*)	715	0.1–1/g	[15]
				manual wash and centrifugation		≥40% by microscopy (IMS/membrane filtration)			qPCR_Taqman (*529 RE*)	163	1/g	
	strawberry (50) lettuce (50)	10^1^ to 10^4^, in 100 mL ddH_2_O for 250 g sample	30 min at RT	wash by manual stirring, filtration through a cellulose ester membrane and centrifugation	1% Tween 80 (100 mL)	NR	US 5× (45 s at 20 Hz, at 2 min intervals	Axy Prep Blood Genomic DNA kit	PCR (*B1 gene*)	115	1000/250 g by immersion using any pre-treatment	[16]
											10/250 g by drip using FT or US	
	blackberries, blueberries, cranberries, raspberries, strawberries (60) herbs ^a^ (35) green onions (35)	5 × 10^3^ *Eimeria papillata*	ON at 4 °C	wash by orbital shaking in bottles for 1 min, centrifugation and Sheather’s solution in the flotation	1 M glycine pH 5.5 (200 mL)	higher for blueberries, leafy herbs and thyme	FT 8× (1 min in N_2_/1 min at 95 °C) and proteinase K	spin columns (QIAamp DNA Mini Kit	qPCR_HRM (*18S rDNA*)	312	For all berry types, 3/g For herbs and green onions, 5–9/g	[17] *
			ON at RT	wash by stomaching in stomacher filter bag, centrifugation and Sheather’s solution in the flotation	elution solution 0.563 mM H_2_Na_2_P_2_O_7_/42.8 mM NaCl (200 mL)	higher for blackberries, cranberries, raspberries and green onions						
				wash by shaking in stomacher filter bag (30 min per side), centrifugation and Sheather’s solution in the flotation								
**immersion**	lettuce (50)	10 to 10^4^, in 2000 mL ddH_2_O	NR	wash by manual stirring, filtration through a cellulose ester membrane and centrifugation	1% Tween 80 (100 mL)	NR	FT 5× (5 min in N_2_/5 min at 65 °C)	spin columns (Axy Prep Blood Genomic DNA	PCR (*B1* gene)	115	≥10/ µL	[18]
									PCR (*529 RE*)	529	≥100/ µL	
	strawberry (50) lettuce (50)	10^1^–10^4^, in 2000 mL ddH_2_O for 250 g sample	stirred 15 s followed by 30 min incubation at RT	wash by manual stirring, filtration through a cellulose ester membrane and centrifugation	1% Tween 80 (100 mL)	NR	NO	Axy Prep Blood Genomic DNA kit	PCR (*529 RE*)	529	1000/250 g by immersion using FT or US	[16]
							FT 5× (5 min in N_2_/5 min at 65 °C)				100/250 g by drip using FT or US	
**NR**	radish (NR) strawberries (NR)	10^1^ to 10^4^	NR	wash in glass beaker by automatic shaker (100 rpm 2 h), flocculation method using CaCO_3_ solution ON, centrifugation	1% Tween 80 (2000 mL)	NR	BB (6.5 m/s for 2 min), chloroform treatment, and proteinase K incubation	FAST-Prep for soil matrix E combined with Genomic Mini Kit	qPCR_Taqman (*B1 gene*)	128	100/single radish 10,000/single strawberries	[19]
**NR**	raspberries (30) blueberries (30)	10 or 50 in 250 µL (sediment)	NA	wash by automatic shaker (300-600 rpm 10 min) and centrifugation	1% Alconox (200 mL)	NA	BB 2× (4 m/s for 60 s)	DNeasy Power Soil	multiplex qPCR_Taqman (*529 RE*)	163	10/250 µL (sediment from 30 g berries)	[20]

Abbreviations: NR = not reported; NA = not applicable; HRM = High Resolution Melting curve analysis * This paper reported spiking with oocyst of the surrogate apicomplexan *Eimeria papillata*; LoD = Limit of Detection; LAMP = Loop-Mediated Isothermal Amplification; RT = room temperature; ON = overnight; FT = Freeze and thaw; BB = Bead-beating; US = ultrasound; IMS = immunomagnetic separation; qPCR = real time PCR; nPCR = nested PCR. ^a^ herbs: cilantro, dill, mint, parsley, thyme ^b^ Stomaching is the process of sample homogenization in a stomacher (homogenizer) apparatus ^#^ different buffers: E-pure water; 3% levulinic acid/3% Sodium dodecyl sulfate; 1 M Glycine pH 5.5; 0.1M PBS pH 7.0; 0.1% Alconox; 1% HCl pH 2/6.4% pepsin ^##^ 4/g with 85.2% (±25.7) success rate; 34/g with 81.5% (±23.1) success rate; 4/g with 85.2 % (±6.4) success rate; 4/g with 74.1 % (±25.7) success rate; 4/g with 77.8 % (±11.1) success rate; 4/g with 92.6 % (±12.8) success rate ^###^ oocyst recovery calculated by (N0/N) × 100 with N and N0, the total number of oocysts or oocyst equivalents recovered and initially inoculated to the matrix respectively.

**Table 2:** Published methods for the molecular detection of *Toxoplasma gondii* in fresh produce that have been used in surveys.

Matrix (Number)	Source (Number)	Amount of Sample Tested	Processing Method	Washing Buffer (mL)	Pre-Treatment before DNA Extraction	DNA Extraction	Detection Method (Target Gene)	Amplicon Size (bp)	LoD (Oocysts)	Sample Positive (%)	Reference
strawberries (60) carrot (46) radish (60) lettuce (50)	retail shops and marketplaces (175) kitchen-gardens and allotments (41)	1 kg strawberries, 0,5 kg carrot, 20 radishes, 1 lettuce	wash in glass beaker? by automatic shaker (100 rpm 2 h), flocculation method using CaCO_3_ solution ON, centrifugation	1% Tween 80 (2000 mL)	BB (6.5 m/s for 2 min)	Chloroform, proteinase K incubation and Genomic Mini Kit	qPCR_Taqman (*B1*)	128	1	21/216 (10% ) 3 radish 9 carrot 9 lettuce	[19] *
arugula/baby arugula (107) kale (44) spinach/baby spinach (387) romaine (113) chard (39) leaf lettuces1 (226) spring mix (124) leafy green mixes (91) other mixes (3)	retail outlets (1171)	35 g	wash by orbital shaker or stomacher (romaine, red or green leafy lettuces only), centrifugation and flotation procedure using Sheather’s solution	1 M glycine pH 5.5 (200 mL)	FT 8× (1 min N_2_/1 min. 95 °C) and proteinase K	QIAamp DNA Micro Kit or Dneasy Blood and Tissue Kit	Multiplex qPCR_HRM (*18S rDNA*)	312	10	3/387 (0.78%) baby spinach	[21]
crisp lettuce (106) regular lettuce (62) chicory (40) rocket (7) parsley (5)	open fairs (77) from producers’ fairs (81), from community fairs (80)	50 g	wash by manual stirring, filtration through a cellulose ester membrane and centrifugation	1% Tween 80 (100 mL)	FT 5× (5 min N_2_/5 min. 65 °C)	Axy Prep Blood Genomic DNA	PCR (*B1*)	115	NR	9/238 (3.8 %) 4 crisp lettuce 1 chicory 1 rocket 1 parsley	[18]*
							PCR (*529 RE*)	529	NR	1 chicory 1 regular lettuce	
RTE mixed salad (curly and escarole lettuce, red radish, rocket salad and carrots) (648)	retail shops (648)	100 g (9 salad packages, 72 pools)	wash by orbital shaker for 15 min at 120 rpm and centrifugation	10X PBS, 0.1% Tween-80, 0.1% SDS and 0.05% antifoam B (200 mL)	FT 15× (1 min N_2_/1 min. 95 °C)	QiAmp Plant Mini Kit	qPCR_HRM (*B1*)	128	NR	0.8%	[22]
lettuce (71) spinach (50) pak choi (34) chinese cabbage (26) rape (22) asparagus (18) chrysanthemum coronarium (16) endive (14) chinese chives (11) cabbage (9) red cabbage (8)	open markets	NR	sample rinsing and Al_2_(SO_4_)_3_ flocculation of washing suspensions	NR (NR)	FT 10× (N_2_/water bath)	TIANamp Micro DNA Ki	qPCR_Taqman (*B1*)	129	1	10/279 (3.6%) 5 lettuce 2 spinach, 1 pak choi 1 red cabbage 1 rape	[23]
carrots (93) slicing cucumbers (109) salads (90) (butterhead lettuce, iceberg lettuce, little gem and lollo lettuce)	9 farms (292)	100 g	wash by automatic shaker for 20 min and centrifugation	Tris–glycine beef extract pH 9.5 (230 mL)	BB (6400 rpm for 60 s)	Power-Soil DNA isolation kit	Triplex qPCR_Taqman (*B1* + *529RE*)	129 (B1) 163 (529 RE) 157 (IAC)	NR	28/292 (9.6%) 7 Carrots 13 cucumbers 8 salads	[24]

Abbreviations: HRM = High Resolution Melting curve analysis; * Also reporting on spiking studies; ON = overnight; FT = Freeze and thaw; BB = Bead-beating; NR = not reported.

**Table 3:** Studies comparing the performance of freeze and thaw cycles vs bead-beating as pre-treatment procedure to DNA extraction from *Toxoplasma gondii* oocysts.

Matrix (Amount)	Spiking Level	Pre-Treatment before DNA Extract	DNA Extraction	Detection (Target Gene)	Limit of Detection (oo)cysts	Reference
faeces (200 mg)	10^1^–10^4^	BB	NucleoSpin Soil using Buffer SL2 + Enhancer SLX	PCR (*529 RE*)	10	[25]
		BB	ZR fecal DNA Kit		10	
		FT 3× (10 min at −20 °C/2 min at RT)	phenol/chloroform extraction (in-house)		100	
water or mussels tissues (NR)	10^0^–10^3^	FT 5× (liquid N_2_/70 °C) +BB (glass beads, 30 s at 4200 rpm) + proteinase K 1 h at 56 °C	spin column	PCR (*529 RE*)	1 (100%) in water and hemolymph	[26]
		vortexing (PowerSoil beads max speed for 10 min) + BB (30 s at 4200 rpm)	PowerSoil™ DNA Isolation Kit		10 (100%) in water and hemolymph, (50%) in dig. glands; 100 (100%) in gills and dig. glands	
water or mussels tissue homogenate (100 µl)	10^0^–10^3^	FT 1× (4 min liquid N_2_/4 min 100 °C)	spin column	nested PCR (*B1*)	100 (100%) with 1, 3 or 6 cycles; 10 (60%) with 1 or 6 cycles; 1 (30%) with 1 cycles	[27]
		FT 3× (4 min liquid N_2_/4 min 100 °C				
		FT 6× (4 min liquid N_2_/4 min 100 °C				

Abbreviations: BB = beat beating; FT = freeze-thaw cycles; RT= room temperature; NR = not reported.

**Table 4:** Conventional PCR assays targeting *B1* gene and *529* RE used for *Toxoplasma gondii* oocysts detection.

Target Gene	Amplicon Size (bp)	LoD (Number of Spiked Oocysts That Provide Positive Amplification)	Primer Pairs	Matrix	Reference
*B1*	115	≥10 oocysts in 250 g of strawberry or 1 lettuce head	B22: 5′-AACGGGCGAGTAGCACCTGAGGAGA-3′ B23: 5′-TGGGTCTACGTCGATGGCATGACAAC-3′	fresh produce	[16]
		10 oocysts		water	[16]
		10 oocysts/µL spiking level		fresh produce	[18]
	194	NR	Oligo1: 5′-GGAACTGCATCCGTTCATGAG-3′ Oligo4: 5′-TCTTTAAAGCGTTCGTGGTC-3′	soil	[36]
		50 tachyzoites /0.5 g soil		soil	[37]
		NR		soil	[34]
		100 oocysts		faeces	[38]
		25 oocysts/30 g soil or ≤1 oocyst/1g soil		soil	[39]
*529 RE*	529	≥100 oocyst in 250 g of strawberry or 1 lettuce head	TOX4: 5′-CTGCAGGGAGGAAGACGAAAGTTG-3′ TOX5: 5′-CTGCAGACAGAGTGCATCTGGATT-3′	Fresh produce	[16]
		10 oocysts		water	[16]
		≥100 oocysts/ µl spiking level		fresh produce	[18]
		NR		faeces	[40]
		1 oocyst in water and mussel hemolymph		mussels	[26]
		100 oocysts in mussel gills and dig. Glands		mussels	[26]
		NR		oysters	[32]
		NR		food	[12]
		5 tachyzoites /0.5 g soil		soil	[37]
		10 oocysts		faeces	[38]
		1–2 oocysts per 200 µL		faeces	[35]
	450	1 oocyst	TOX-8: 5′-CCCAGCTGCGTCTGTCGGGAT-3′ TOX-5: 5′-GACGTCTGTGTCACGTAGACCTAAG-3′	faeces	[38]
		10 oocysts/200 mg feces		faeces	[25]
	529 and 450	NR	TOX4/TOX5 and TOX-8/TOX-5	faeces	[41]
	134	NR	TOXO-F: 5′ AGGCGAGGGTGAGGATGA-3′ TOXO-R: 5′-TCGTCTCGTCTGGATCGCAT-3’	soil	[34]
		NR		soil	[36]

**Table 5:** qPCR-based assay and targets used for *Toxoplasma gondii* oocyst detection in reviewed literature.

Type of qPCR	N° of Studies	Target Gene	N° of Studies
MC/HRMC	9	*B1*	4
		*529RE*	1
		*18SrDNA*	3
		*B1* and *529RE*	1
Taqman	26	*B1*	7
		*529RE*	16
		*ITS1*	1
		*18SrDNA*	1
		*B1* and *18SrDNA*	1
FRET	1	*529RE*	1
HRM and FRET	1	*B1* and *529RE*	1
MC and Taqman	1	*B1*	1
**Total**	**38**		**38**

Abbreviations: HRMC = high resolution melting curve; FRET = Fluorescence Resonance Energy Transfer; MC = melting curve.

**Table 6:** qPCR Taqman assays targeting *B1* gene and *529* RE used for *Toxoplasma gondii* oocysts detection.

Target	Amplicon Size (bp)	IAC (Y/N)	Primer Sequence	Analytical Specificity	Analytical Sensitivity	LoD (Oocysts/g)	LoQ	Matrix	Reference
*529 RE*	169	N	Tox-9F: 5′-AGGAGAGATATCAGGACTGTAG-3′ Tox-11R: 5′-GCGTCGTCTCGTCTAGATCG-3′ Tox-TP1: 5′-6-FAM-CCGGCTTGGCTGCTTTTCCT-BHQ1-3′	[14]	90–100%	5	NR	mussel hemolymph	[28]
					100 oocysts (90%) 10 oocyst (30%)	10–100		mussel tissue	
		Y	Tox-9F; Tox-11R and Tox-TP1	NR	100% 2000 oocysts/g	200	NR	soil	[44]
	164	Y ^a^	Tox-9F; Tox-11R and Tox-TP1	1 with 95% CI of 0.69–1.00	100% (Crypto or Neospora)	50 fg/µL	NR	water	[45]
		N	Tox-9F; Tox-11R and Tox-TP1	NR	NR	1–10 in water and hemolymph	NR	hemolymph and mussel tissue	[26]
	163	Y ^b^	Tox-9F; Tox-11R and Tox-TP1 5’-HEX	NR	NR	NR	NR	fresh produce	[24] ^f^
				NR	NR	NR	NR	water	
	162	N	Tox-9F; Tox-11R and Tox-TP1 5’-Cy5	In silico 100%	NR	0.3	NR	raspberries blueberries	[20]
		N	Tox-9F; Tox-11R and Tox-TP1	100% (test on parasites, mammalian and plant DNA)	50–100 oocysts (100%) 25 oocysts (83%)	0.5		baby lettuce	[14]
	81	Y	[42] Toxo-P 5’-Cy3-ACGCTTTCCTCGTGGTGATGGCG-BHQ2-3’	NR	NR	0.1–1	5 oocyst/5 g	mussel	[29]
				NR	NR	10–50	100 oocysts	haemolymph	
		N	[42]	NR	NR	NR	NR	faeces	[46]
		N	[42]; Toxo-P5′	NR	NR	NR	NR	mussels	[47]
				NR	NR	NR	NR	water	
		N	[42]	NR	NR	NR	NR	soil	[48]
		N	[42]; Toxo-P5′	NR	NR	NR	NR	faeces	[49]
		N	[42]; Toxo-P5′	NR	NR	<1	NR	basil leaves raspberries	[13]
		N N	[42]; Toxo-P5′	NR	NR	NR	NR	hemolymph and mussel tissue	[50]
				NR	NR	NR	NR	water	
		N	[42]	NR [51]	Reported in [42]	10-100	NR	soil	[52]
		N	FOR_5′-AGAGACACCGGAATGCGATCT-3′ REV_5′-CCCTCTTCTCCACTCTTCAATTCT-3′ Probe 5′-6FAM-ACGCTTTCCTCGTGGTGATGGGG-3´TAMRA	NR	NR	10-100	NR	soil	[42]
		N	[42] Probe Tox-HP-1 GAGTCGGAGAGGGAGAAGATGTT-FL Probe Tox-HP-2 Red 640-CCGGCTTGGCTGCTTTTCCTG-Ph	NR	NR	NR	NR	faeces	[53]
*B1*	129	Y ^c^	[54]	NR	NR	NR	NR	fresh produce	[23]
		Y ^b^	[54] ToxB-69p 5′-FAM-ACCGCGAGATGCACCCGCA- BHQ -3′	NR	NR	NR	NR	fresh produce	[24] ^f^
				NR	NR	NR	NR	water	
			ToxB-41: 5′′-TCGAAGCTGAGATGCTCAAAGTC-3′ ToxB-169 5′′-AATCCACGTCTGGGAAGAACTC-3′ 5′′-FAM-ACCGCGAGATGCACCCGCA TAMRA-3′	tested by sequencing	10 molecules of plasmid	100	NR	fruits and vegetables	[19]
	98	Y ^d^	Toxo-F 5′-TCCCCTCTGCTGGCGAAAAGT-3′ Toxo-R 5′-AGCGTTCGTGGTCAACTATCGATTG-3′ probe V5′-FAM-TCTGTGCAACTTTGGTGTATTCGCAG-3′ TAMRA	NR	NR	NR	NR	water	[43]
		N	[43] Toxo-F; Toxo-R and probe V5′	NR	NR	NR	1	water	[55]
				NR	NR	NR	250	faeces	
		Y (N.S.)	[43] Toxo-F; Toxo-R and probe V5′	NR	NR	NR	NR	water	[54]
	62	N	TX2-F 5″-CTAGTATCGTGCGGCAATGTG-3′ TX2-R 5′-GGCAGCGTCTCTTCCTCTTTT-3′ TX2M1 5″-(6-FAM)-CCACCTCGCCTCTTGG-(NFQ-MGB)-3′	NR	NR	5 genomic copies/µL	50 genomic copies/µL	water	[31]
*18S rRNA*	NR	N	[56] Tox18-213f 5′-CCGGTGGTCCTCAGGTGAT-3′ Tox18-332r 5′-TGCCACGGTAGTCCAATACAGTA-3′ Tox18-249p 5′-FAM-ATCGCGTTGACTTCGGTCTGCGAC-TAMRA-3′	NR	NR	NR	NR	water	[57]
	120	Y ^e^	Tox18-213f; Tox18-332r and Tox18-249p	100% (various parasites tested)	NR	10 molecules	NR	hemolymph and mussels	[58]
*ITS1*	NR	N	For 5′-GATTTGCATTCAAGAAGCGTGATAGTA-3′ Rev 5′-AGTTTAGGAAGCAATCTGAAAGCACATC-3′ Probe 5′-/-TET/-CTGCGCTGC/ZEN/TTCCAATATTGG-/-IABkFQ-/-3′	NR	NR	NR	NR	oysters	[59]

Abbreviations: LoQ = Limit of Quantification; IAC = internal amplification control; NR = not reported a competitive IAC as described in [60]. ^a,b^, Artificial DNA sequence; ^c^, *Acanthamoeba* spp. *18SrRNA* gene (180 bp fragment); ^d^, artificial: part of *B1* gene in plasmid; ^e^, ssrRNA of *Mytilus galloprovincialis* (Myt18); ^f^, triplex qPCR consisting of *B1* and *529 RE* as detection targets and artificial IAC.

## Data Availability

No new data were created or analyzed in this study. Data sharing is not applicable to this article.

## Supplementary Materials

The following are available online at https://www.mdpi.com/2076-2607/9/1/167/s1, Supplementary File S1: Excel tables with papers list and extracted data.
